# Biochemical simulation of mutation synthesis and repair during SARS-CoV-2 RNA polymerization

**DOI:** 10.1016/j.virol.2024.110255

**Published:** 2024-10-01

**Authors:** Adrian Oo, Zhenhang Chen, Dongdong Cao, Young-Jae Cho, Bo Liang, Raymond F. Schinazi, Baek Kim

**Affiliations:** a Department of Pediatrics, School of Medicine, Emory University, Atlanta, GA, 30322, USA; b Department of Biochemistry, School of Medicine, Emory University, Atlanta, GA, 30329, USA; c Center for ViroScience and Cure, Children’s Healthcare of Atlanta, GA, 30322, USA

## Abstract

We biochemically simulated the mutation synthesis process of SARS-CoV-2 RNA-dependent RNA polymerase (RdRp) complex (nsp7/nsp8/nsp12) involving two sequential mechanistic steps that occur during genomic replication: misinsertion (incorporation of incorrect nucleotides) and mismatch extension. Then, we also simulated mismatch repair process catalyzed by the viral nsp10/nsp14 ExoN complex. In these mechanistic simulations, while SARS-CoV-2 RdRp displays efficient mutation synthesis capability, the viral ExoN complex was able to effectively repair the mismatch primers generated during the mutation synthesis. Also, we observed that the delayed RNA synthesis induced by mutation synthesis process was rescued by the viral ExoN activity. Collectively, our biochemical simulations suggest that SARS-CoV-2 ExoN complex may contribute to both maintenance of proper viral genetic diversity levels and successful completion of the viral large RNA genome replication by removing mismatches generated by the viral RdRp.

## 1. Introduction

Human severe acute respiratory syndrome coronavirus 2 (SARS-CoV-2) was first identified in January 2020 as a novel coronavirus associated with an initial outbreak in Wuhan, China (Spiteri et al., 2020). Thenceforward, infection with this single-stranded, positive-sense RNA virus has resulted in more than 600 million cases of coronavirus disease 2019 (COVID-19), including about 6 million deaths in an ongoing global pandemic. As other viruses of the *Coronaviridae* family do, SARS-CoV-2 utilizes the RNA-dependent RNA polymerase (RdRp) complex for the replication of its exceptionally large size viral RNA genome (Grellet et al., 2022). The viral RdRp complex consists of three non-structural proteins (nsps), namely nsp12 which consists of the polymerase catalytic subunit, as well as nsp7 and nsp8 working in tandem as a complex with nsp12 as accessory subunits which are required for effective RNA syntheses (Wilamowski et al., 2021). Cryo-electron microscopy analysis has shown that the architecture of this viral RdRp complex shares close similarities with its predecessor, SARS-CoV (Hillen et al., 2020). The N-terminal of the catalytic nsp12 subunit is comprised of a nidovirus-specific RdRp-associated nucleotidyltransferase (NiRAN) domain, which is connected with the right-hand structure RdRp active site via an interface domain (Gao et al., 2020; Yin et al., 2020). The highly-conserved nsp7/nsp8 pair binds to the thumb domain of nsp12, while an additional 13-amino acid residue at the N-terminal of nsp8 binds to the finger structure (Gao et al., 2020).

SARS-CoV-2 RdRp complex has been a crucial target for antiviral drug development especially nucleoside analogs, due to its primary role in the virus replication (Vicenti et al., 2021). Biochemical analyses have demonstrated effective inhibition of SARS-CoV-2 RdRp activities via delayed chain-termination or chain-termination by remdesivir or sofosbuvir, respectively (Bravo et al., 2021; Sacramento et al., 2021). On the other hand, molnupiravir and favipiravir are actively incorporated by SARS-CoV-2 RdRp during RNA syntheses, resulting in viral suppression via lethal mutagenesis (Kabinger et al., 2021; Zhao and Zhong, 2021). Indeed, remdesivir and molnupiravir have been approved by the FDA for the clinical treatment of COVID-19 patients (Ashour et al., 2022; Panda et al., 2021). RNA viruses are generally known to exhibit higher mutation rates (as high as 10^−3^ base misincorporation per nucleotide) than their DNA counterparts (10^−8^ to 10^−6^ error frequency), and SARS-CoV-2 was no exception (Domingo et al., 2021; Peck and Lauring, 2018). The mutagenic nature of SARS-CoV-2 (≈6.7 × 10^−4^ substitution per site per year) is strongly associated with its error-prone RdRp, in addition to the rapid turnover rate of new infectious progeny (Amicone et al., 2022; Sender et al., 2021; Wang et al., 2022a). As random genetic mutations commonly occur during every viral replication cycle, new variants have consistently emerged due to their enhanced replication fitness (Cao et al., 2022; Liu et al., 2022).

While SARS-CoV-2 RdRp enzyme kinetics were previously shown to function in three distinct catalytic pathways, namely nucleotide-addition burst, slow nucleotide addition involving ≤1 s pauses, or very slow nucleotide addition with 1–5 s pauses, the enzyme’s fundamental fidelity kinetics have not been reported thus far (Bera et al., 2021). We have previously established biochemical assays to study the enzyme fidelity properties of influenza A virus (IAV) RNA polymerase as well as multiple reverse transcriptases (RT) including HIV-1 and MuLV RTs (Aggarwal et al., 2010; Skasko et al., 2005). Unlike SARS-CoV-2, both IAV and HIV-1 do not have the exonuclease (ExoN) proofreading capability (Al Farroukh et al., 2022; Boivin et al., 2010; Cuevas et al., 2015; Liu et al., 2021; Roberts et al., 1988).

Genomic replication of SARS-CoV-2 is closely mitigated by the viral ExoN activity found within the N-terminal catalytic site of its 60 kDa nsp14. Nucleoside analog inhibitor or any misincorporated nucleotide would be excised and removed from the growing RNA chain via the 3′–5′ ExoN activity of nsp14, which functions as a complex with its active site-stabilizing cofactor, nsp10 (Bouvet et al., 2012; Liu et al., 2021; Moeller et al., 2022; Saramago et al., 2021). The vital role of nsp14 in ensuring faithful replication of SARS-CoV-2 is evident as alteration in its coding sequence is associated with enhanced mutation rates across the SARS-CoV-2 genome, whereas a loss-of-function double mutation (D90A/E92A) in the protein abrogates viral RNA synthesis (Eskier et al., 2020; Ogando et al., 2020). Separate studies have also reported that other betacoronaviruses such as mouse hepatitis virus (MHV) and SARS-CoV, were more susceptible towards the otherwise resistant mutagenic molecules such as 5-fluorouracil, when they lose their ExoN activities (Graepel et al., 2017; Smith et al., 2013).

Here, we report a biochemical system that simulates both mutation synthesis by SARS-CoV-2 RdRp during RNA polymerization and repair processes catalyzed by the viral ExoN complex. The two sequential steps that occur during mutation synthesis by the RdRp were investigated in the presence and absence of the viral ExoN complex. These biochemical simulations support two potential mechanistic roles of the SARS-CoV-2 ExoN complex in both maintenance of the proper viral mutagenesis levels and successful replication of the exceptionally large size viral RNA genomes.

## 2. Materials and methods

### Proteins purification:

SARS-CoV-2 nsp7, nsp8 and nsp12 proteins were expressed and purified as described previously (Zandi et al., 2020). Plasmid vectors for SARS-CoV-2 nsp10 and nsp14 expression were synthesized with a 10xHis-tag and a 10xHis-3 Strep tag, followed by a Tev cleavage site at the N-terminus of either gene, respectively. The nsp14 vector was then employed as a template for the generation of the E191A/D273A double mutant, which were obtained through overlap PCR with individual primer sets. The integrity of the construct was confirmed via sequencing. Plasmid containing nsp10, nsp14 WT or nsp14 E191A/D273A double mutant, was transformed into the BL21 (DE3) E. coli strain. A single colony was inoculated in LB medium supplied with ampicillin or kanamycin at 37 °C with overnight shaking at 200 rpm. The preculture was then transferred into LB medium for large scale expression. When OD_600nm_ was between 0.6 and 0.8, protein expression was induced with 1 mM isopropyl β-D-1-thiogalactopyranoside (IPTG) at 16 °C overnight. The cells were pelleted through centrifugation at 4000 rpm for 30 min. For nsp14 WT or E191A/D273A double mutant, the cell pellets were resuspended with buffer **A1** (50 mM Tris-HCl, pH 8.5; 300 mM NaCl; 10 % glycerol), and disrupted by sonication. Cell debris were removed by centrifugation at 18,000 rpm for 40 min. The supernatant was loaded on a gravity Ni-NTA column (HisPur Ni-NTA Resin, Thermo Scientific) that was pre-equilibrated with buffer **A1**. Contaminants and unbound proteins were removed by washing the column with 15 column volumes of buffer **A1**, supplemented with 50 mM imidazole. The target protein was eluted with buffer **A1** containing 100–300 mM imidazole. Fractions containing nsp14 were pooled and subjected to Tev protease digestion with a simultaneous dialysis against buffer **A1** at 4 °C overnight, after which the sample was ran through another gravity Ni-NTA column (HisPur Ni-NTA Resin, Thermo Scientific). The flow through sample was dialyzed to buffer-exchange to buffer **B** (25 mM HEPES pH 8.0; 50 mM NaCl; 0.5 mM TCEP) and purified further with a HiTrap Heparin HP column (Cytiva) using buffer **B** and buffer **C** (25 mM HEPES pH 8.0; 1 M NaCl; 0.5 mM TCEP). The sample was further polished by size-exclusion chromatography using a Superdex 200 Increase 10/300 GL column (Cytiva) which was pre-equilibrated with buffer **D** (25 mM HEPES pH 8.0; 150 mM NaCl; 0.5 mM TCEP). The peak fractions containing nsp14 were pooled, concentrated, flash-frozen in liquid nitrogen, and stored at −80 °C until use for downstream applications. The protein purity was verified using SDS-PAGE. Nsp10 was purified with the same procedures as nsp14 as described above, except that buffer **A1** and the heparin column were replaced with buffer **A2** (40 mM Tris-HCl, pH 8.0; 500 mM NaCl; 10 % glycerol), a HiTrap Capto Q ImpRes column (Cytiva) and a Superdex 75 10/300 GL column (Cytiva).

### Biochemical SARS-CoV-2 RdRp and ExoN assays:

SARS-CoV-2 RdRp complex (0.1 μM, also termed as 1X) was prepared by incubating a mixture of purified nsp7, nsp8 and nsp12 (Fig. S1). The nsp8 and nsp7 components were first pre-mixed in equimolar amounts for 15 min on ice, prior to mixing with nsp12 for another 15 min on ice. The final complex consisted of nsp12:nsp8-nsp7 at a 1:4 ratio, with 0.1 μM concentration of nsp12. *In vitro* RNA syntheses were conducted at 37 °C for 30 min with the RdRp complex, in the presence of 25 mM TRIS-HCl (pH 8.0), nonradioactive NTPs (50 μM ATP, CTP, UTP; 25 μM GTP; unless otherwise described in certain assays), 2 μM of 5’ ^32^P-labeled RNA primer-template (For ^32^P α-GTP incorporation setup: 0.1 μM ^32^P α-GTP, 200 μM RNA primer, 2 μM RNA template) and 5 mM MnCl_2_. The RdRp reactions were terminated by adding formamide containing 40 mM EDTA and heated at 95 °C for 10 min. On the other hand, SARS-CoV-2 ExoN complex (0.1 μM) was prepared by incubating a mixture of nsp10 and nsp14 (Fig. 4A) on ice for 15 min. RdRp products or radiolabelled RNA primer-templates were incubated with ExoN complex in the presence of 25 mM TRIS-HCl (pH 8.0), 1 mM dithiothreitol and 5 mM MgCl_2_ at 37 °C for 20 min. Each reaction product was then resolved on 20% polyacrylamide-urea denaturing gels (National Diagnostics). Gel visualization and analysis were performed using the Amersham Typhoon IP (Cytiva) and ImageQuant TL 8.2 (Cytiva), respectively.

## 3. Results

### RNA polymerization with biased rNTP pools by SARS-CoV-2 RdRp:

**Fig. 1.**
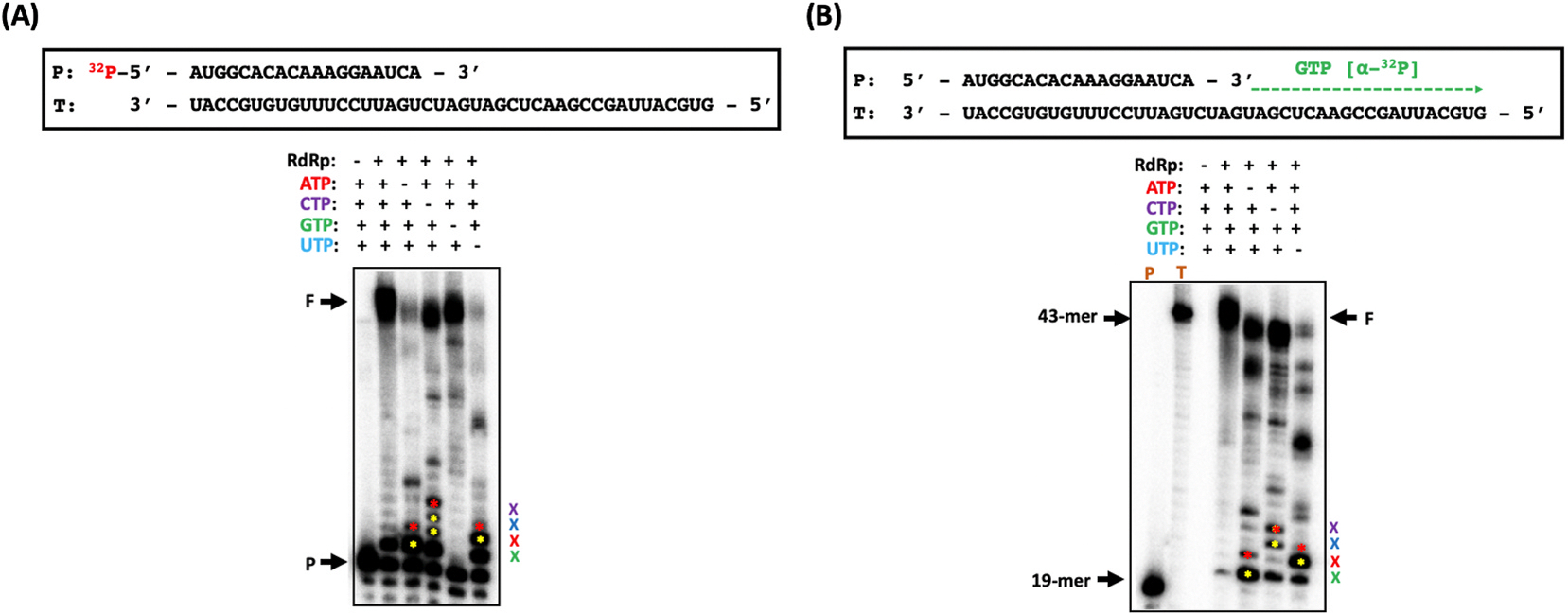
Nucleotide misincorporation by SARS-CoV-2 RdRp during RNA syntheses. (A) A 5′ end ^32^P-labeled RNA primer-template was subjected to polymerization using the purified RdRp complex in the presence of all nonradioactive rNTPs or in the absence of one of the selected rNTP. Pause sites were marked as “*” in yellow for sites prior to the actual nucleotide incorporation site, and red for 3′end mismatch-associated pauses, whereas the site where the missing rNTP was supposed to be incorporated was denoted as “X” (ATP in red; CTP in magenta; GTP in green; UTP in blue). The full-length 43-mer RNA products were marked as *“F”* whereas the unextended primer was labeled as *“P”*. (B) Similar reaction was set up as (A) apart from α-^32^P GTP was being incorporated as the primer was being extended. (−) GTP condition was not tested as the radioactive rNTP used was a GTP.

We established a primer extension assay of SARS-CoV-2 RdRp using a 19-mer RNA primer annealed to a 43-mer RNA template (see boxes in Fig. 1A and B), and the full extension of the 19-mer primer by the RdRp complex will generate a 43-mer RNA product (see “F” in Fig. 1A and B), which can be visualized in denaturing gels. We have employed two different methods to visualize the RNA products: 1) ^32^P pre-labeling of the 19-mer primer at its 5’ end (“P”), which was then annealed to the 43-mer RNA template (Fig. 1A). To our knowledge, this is the first report on SARS-CoV-2 RdRp assay utilizing this method, which allows us to visualize the entire RNA primer products during the polymerization reactions; 2) Radioactive labeling of the RNA products via incorporation of ^32^P α-GTP during primer extension (Fig. 1B). This published method visualizes the products only when ^32^P α-GTP molecules are incorporated into the extended RNA strand (Gordon et al., 2021; Zandi et al., 2020). As shown in Fig. 1A and B, when all four rNTPs were present in the SARS-CoV-2 polymerization reactions, both visualization methods displayed the fully extended 43-mer RNA products (see “F”), confirming the establishment of the biochemical RNA primer extension reaction of SARS-CoV-2 RdRp. In addition, these reaction conditions were established based on both reaction time and RdRp concentration that generate linear amounts of full-length RNA products (Figs. S2 and S3).

Next, we employed these RNA primer extension reactions to monitor the overall pattern of mutation synthesis during RNA polymerization by SARS-CoV-2 RdRp. For this, we conducted the primer extension reactions with biased rNTP pools harboring only three kinds of rNTPs (or missing one of the four rNTPs). We and others have extensively used this type of assay, known as “misincorporation assay” for investigating the enzyme fidelity of various DNA and RNA polymerases (Aggarwal et al., 2010; Diamond et al., 2003; Scull et al., 2019; Skasko et al., 2005; Vaisman et al., 2000). Under the biased rNTP pool conditions, the primer extension initially paused at nucleotide position(s) on the template sequence (marked as “yellow *“) prior to the site where the missing rNTP was supposed to be incorporated (denoted as “X”) (Fig. 1A and B). Furthermore, the pauses force the RdRp to incorporate incorrect rNTPs, generating mismatches at the sites where the missing rNTPs were supposed to be incorporated. Since the mismatches are poorly extended, compared to normal/matched sites, another type of pauses due to the unextended mismatches at the 3’ ends of the growing RNA products can be often visualized (see “red *“). Finally, the RdRp will extend the mismatches and continue the RNA synthesis, completing a single mutation synthesis. Importantly, as the two different pauses of the RNA synthesis induced by the biased substrate pools can diminish the overall amounts of the full-length product, compared to the reactions in the presence of all four natural substrates, the misincorporation assay with biased substrate pools has been used to monitor the overall enzyme fidelity of DNA and RNA polymerases (Aggarwal et al., 2010; Diamond et al., 2003).

In addition, as shown in the reactions with biased rNTP pools (Fig. 1A and B), SARS-CoV-2 RdRp displayed reduced full-length products, compared to the control reactions with all four rNTPs. Interestingly, although the frequency of each rNTP incorporation sites is relatively similar on the template sequence, the efficiency of SARS-CoV-2 RdRp in generating a possibly mutated full-length 43-mer RNA product was more sensitive towards the deletion of ATP or UTP, and the CTP deletion in the reaction appeared to be more tolerant, indicating that SARS-CoV-2 RdRp tends to generate mutations at the G sites of the template, but not at the A sites within the template. In fact, our previous study with influenza virus RdRp complex also reported varying misincorporation specificity, depending on the rNTP deleted in the polymerization reactions (Aggarwal et al., 2010). It is also important to take note that the nucleotide misincorporation patterns and efficiencies are also affected by the surrounding template sequence.

**Fig. 2.**
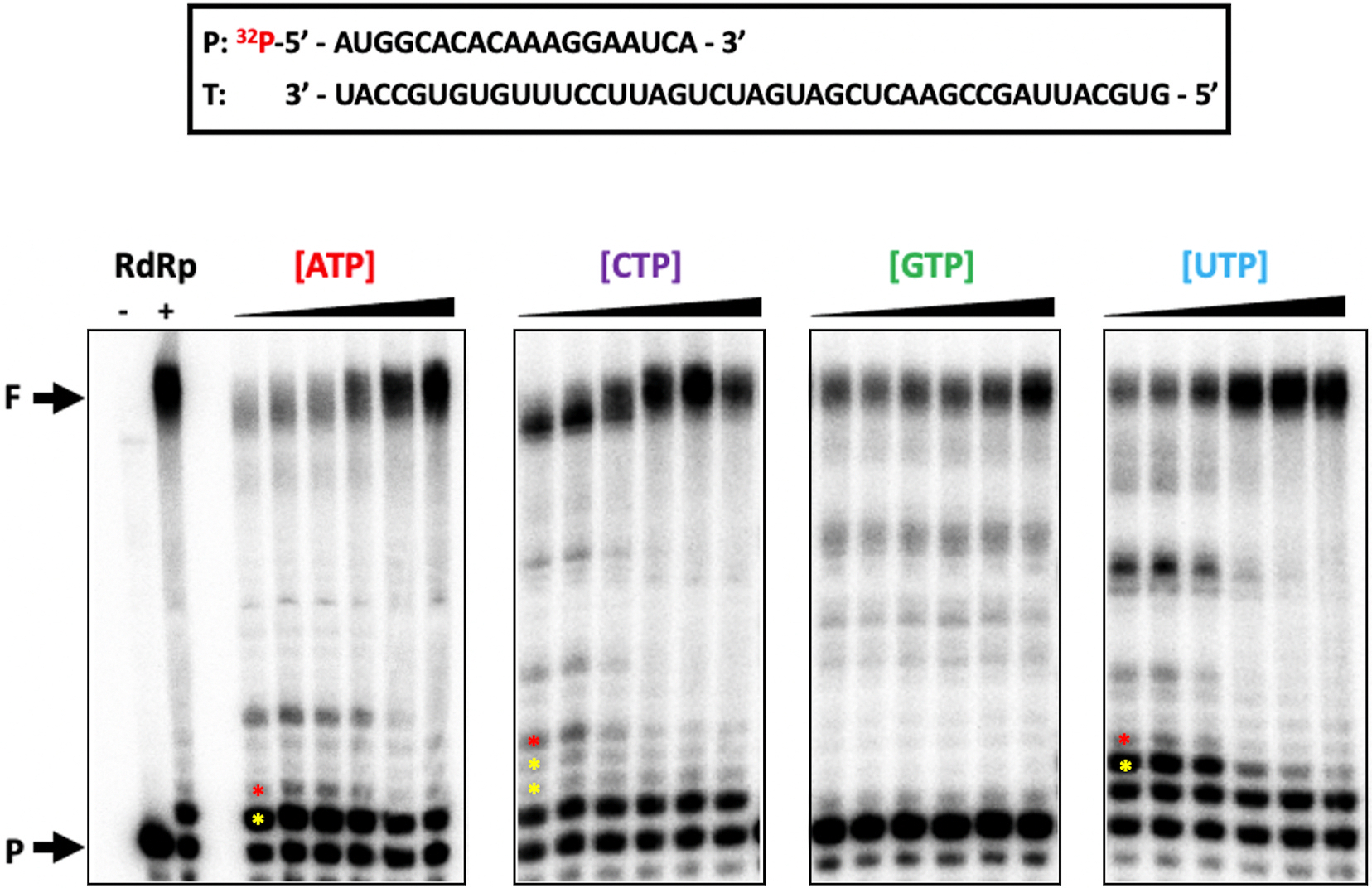
NTP-concentration dependent RNA primer extension by SARS-CoV-2 RdRp. A 5′ end ^32^P-labeled RNA primer-template was subjected to polymerization using the purified RdRp complex in increasing concentrations of selected nonradioactive NTPs (10-fold increments from 0.05 nM to 5 μM for ATP, CTP and UTP; 0.025 nM to 2.5 μM for GTP). RdRp (+) control was 50 μM nonradioactive ATP, CTP and UTP as well as 25 μM GTP. The full-length 43-mer RNA products were marked as *“F”* whereas the unextended primer was labeled as *“P”*. Pause sites were marked as “*” in yellow for sites prior to the actual nucleotide incorporation site, and red for 3′end mismatch-associated pauses.

Next, we conducted a ‘reverse’ misincorporation reactions, whereby increasing concentrations of the missing rNTP were added back into the reactions (Fig. 2). The added rNTPs were expected to remove the pause sites (”*“) induced by the absence of respective missing substrates. As shown in Fig. 2, when we added each of the missing rNTPs back into the reactions with gradually increasing concentrations, the synthesis of the full-length products, which was delayed by the pause sites, gradually increased. Indeed, the adding-back of the increasing concentrations of the missing rNTPs also gradually removed the pause sites (”*“), confirming that those pause sites observed in the misincorporation assay (Fig. 1A and B) were generated by the stalling of the SARS-CoV-2 RdRp during RNA synthesis. Furthermore, consistently observed initial pauses at +1 sites across every RNA elongation reaction indicate the abortive initiation nature of SARS-CoV-2 RdRp, whereby the abortive products were prematurely released from the replication complex following elongation of the first nucleotide. However, it should be noted that the degree of abortive initiation of similar viral enzymes may vary across different nucleotide sequences as it is commonly template-dependent (Hsu et al., 2003).

### Mismatched primer extension by SARS-CoV-2 RdRp:

**Fig. 3.**
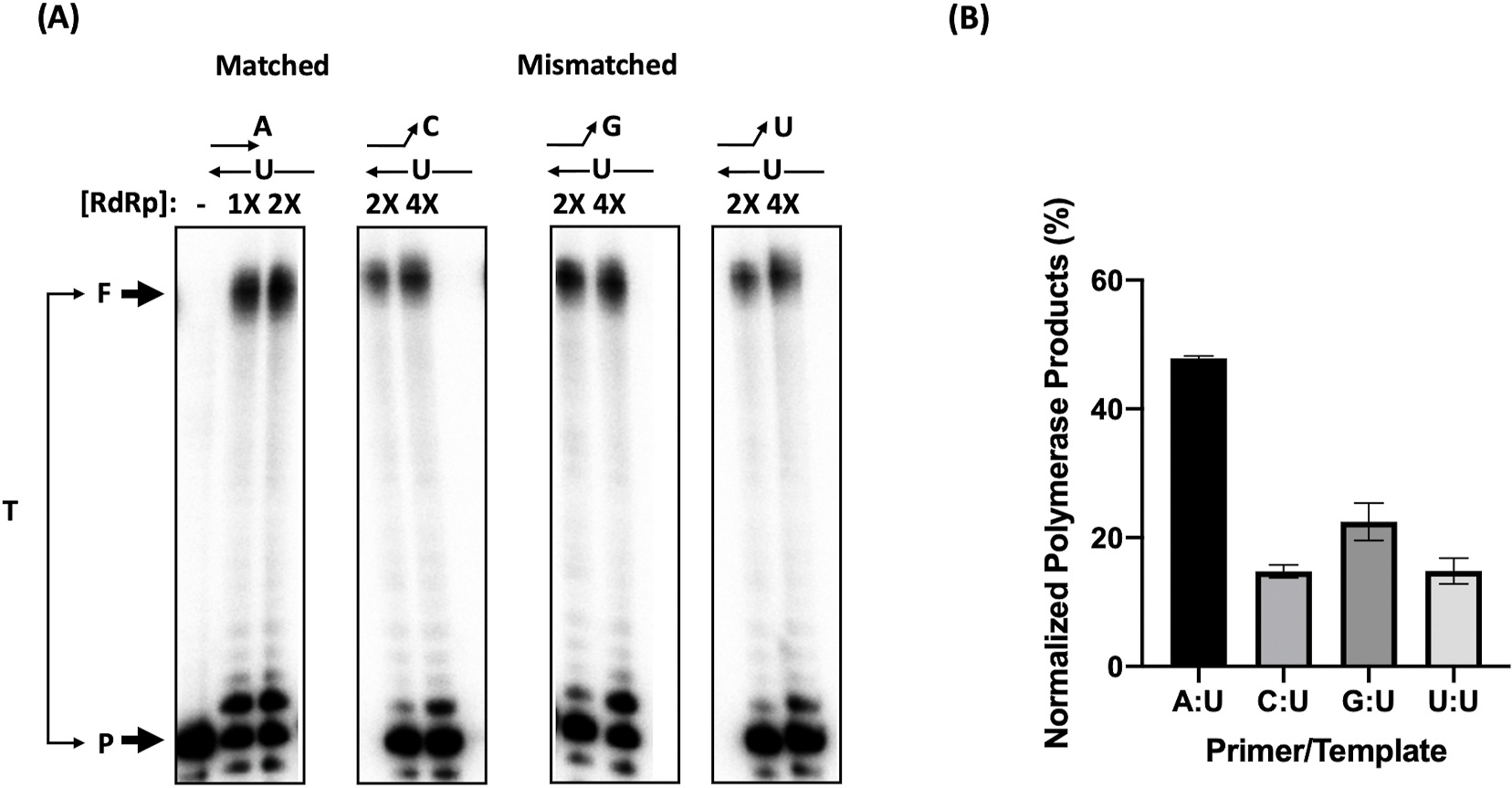
Extension of RNA primers with matched or mismatched 3′ ends by SARS-CoV-2 RdRp. (A) RNA transcription by SARS-CoV-2 RdRp complex was performed using similar 5′ end ^32^P-labeled RNA primer-template as Figs. 1B and 2, with or without a mismatched nucleotide at the 3′ end of the primer. Each primer-template was extended with two concentrations of the RdRp complex (1X and 2X for matched; 2X and 4X for mismatched primer-template sets). The amount of full-length RNA products *“F”* extended from the primers *“P”* for each reaction was normalized to the total radiation signal *“T”* quantified for respective lanes. (B) Graphical representation of normalized polymerase products extended using 2X [RdRp] for each primer-template set as illustrated in (A).

For the mutation synthesis during enzymatic DNA/RNA polymerization, two mechanistically different sequential steps need to occur: 1) misinsertion (incorporation of an incorrect nucleotide), which generates a mismatch primer at the 3′ end of the growing primer, and 2) mismatch extension which requires the incorporation of a correct nucleotide from the 3′ mismatch. As described earlier in our nucleotide misincorporation assay (Fig. 1A and B), SARS-CoV-2 RdRp was still able to generate some levels of the full-length products even in the absence of one rNTP during RNA synthesis. This suggests that SARS CoV-2 RdRp actively extends the mismatch primers during RNA syntheses. Hence, we next simulated the efficiency of the viral enzyme to promote RNA strand extension from mismatched 3′ ends generated by misincorporated nucleotides. Here, we performed the SARS-CoV-2 RdRp assays using RNA primer-template sets with (C:U, G:U, and U:U) or without (A:U) mismatched 3′ end on the extending primers. It is important to note that the amounts of the full length product in these polymerase reactions are linear to the RdRp amounts and incubation time under this reaction condition as described earlier (Figs. S2 and S3). As shown in Fig. 3A and B, the viral enzyme exhibits lower primer extension capacity from mismatched primers even when higher concentration of the enzyme (2x) was used in comparison to that of matched primer-template (1x). Relatively similar potency in primer extension between the matched and mismatched primer-template sets were only observed when the RdRp concentration was further increased (4x) for RNA syntheses in the presence of a mismatch (Fig. 3A). When the total full length products in these reactions were normalized (Fig. 3B), approximately 70% reduction in primer extension capabilities were determined with the C:U and U:U mismatched primers, SARS-CoV-2 RdRp was slightly more efficient in extending the G:U mismatched primer (≈53% reduction relative to matched A:U primer). However, it is worth to take note that albeit the lower primer extension efficiency, SARS-CoV-2 RdRp was indeed still capable of extending the RNA strand from a mismatched 3’ end of the primer and produced significant levels of the full-length products. This supports the possibility that SARS-CoV-2 RdRp harbors a relatively high mismatch extension capability.

### SARS-CoV-2 ExoN repairs mismatched ends of primers:

**Fig. 4.**
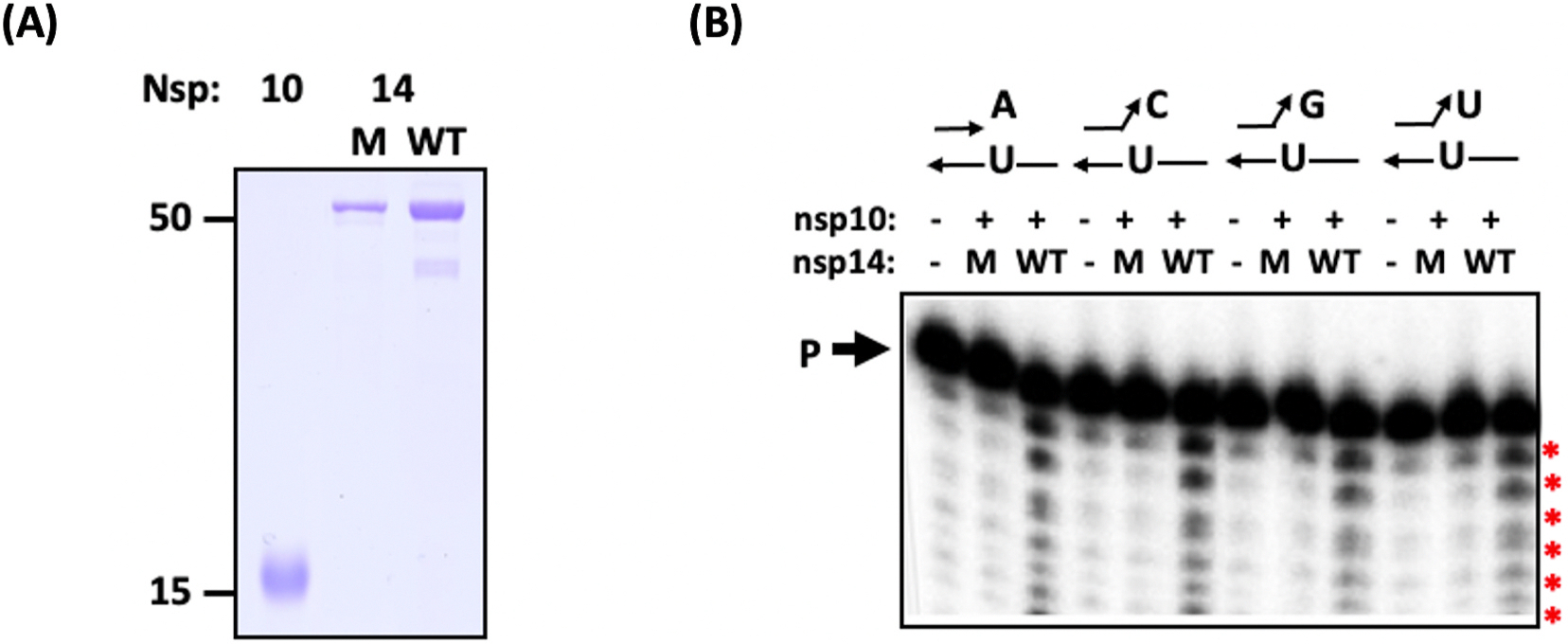
ExoN degradation activity of SARS-CoV-2 nsp14 in complex with nsp10. (A) SDS-PAGE of purified SARS-CoV-2 nsp10 as well as nsp14 WT and E191A/D273A double mutant *“M”*. (B) ExoN activities of SARS-CoV-2 WT or E191A/D273A double mutant nsp14, in complex with nsp10 on RNA primer-template sets with matched or mismatched nucleotide at the 3′ ends of the primers. 5′ end ^32^P-labeled RNA primer-template sets were labeled as *“P”* whereas ExoN-degraded RNA fragments were marked in red asterisks.

SARS-CoV-2 possesses the proofreading ExoN that was previously proposed to repair misincorporated nucleotide or nucleoside analog inhibitor (Liu et al., 2021; Moeller et al., 2022). The viral nsp14 is a multifunctional protein with an N-terminal ExoN domain and a C-terminal N7-MTase domain. It has been previously reported that the viral nsp10-nsp14 complex exhibits 3′–5′ ExoN activity on dsRNA with or without mismatch 3′ ends (Moeller et al., 2022). Here, we tested whether nsp10-nsp14 complex can remove the 3′ end mismatches used in Fig. 3. For this test, we purified nsp10 and nsp14 (Fig. 4A), and the nsp10-nsp14 complex was pre-formed prior to the exonuclease assay. We also constructed and purified enzymatically inactive nsp14 mutant protein containing E191A/D273A active site mutations. As shown in Fig. 4B, we have observed ExoN degradation activities on our matched (A:U) as well as each of the mismatched (C:U, G:U, U:U) RNA primer: templates, in the presence of WT nsp10-nsp14 complex. On the other hand, ExoN activities were not detected with the E191A/D273A double mutant (M) nsp14. The data shown in Fig. 4 suggests that nsp10-nsp14 complex may help mitigate the relatively high efficiency of mismatch extension capability of SARS-CoV-2 in order to minimize the overproduction of viral genomic mutations during viral replication.

### Nsp10-nsp14 complex rescues reduced RNA synthesis induced by mismatches:

**Fig. 5.**
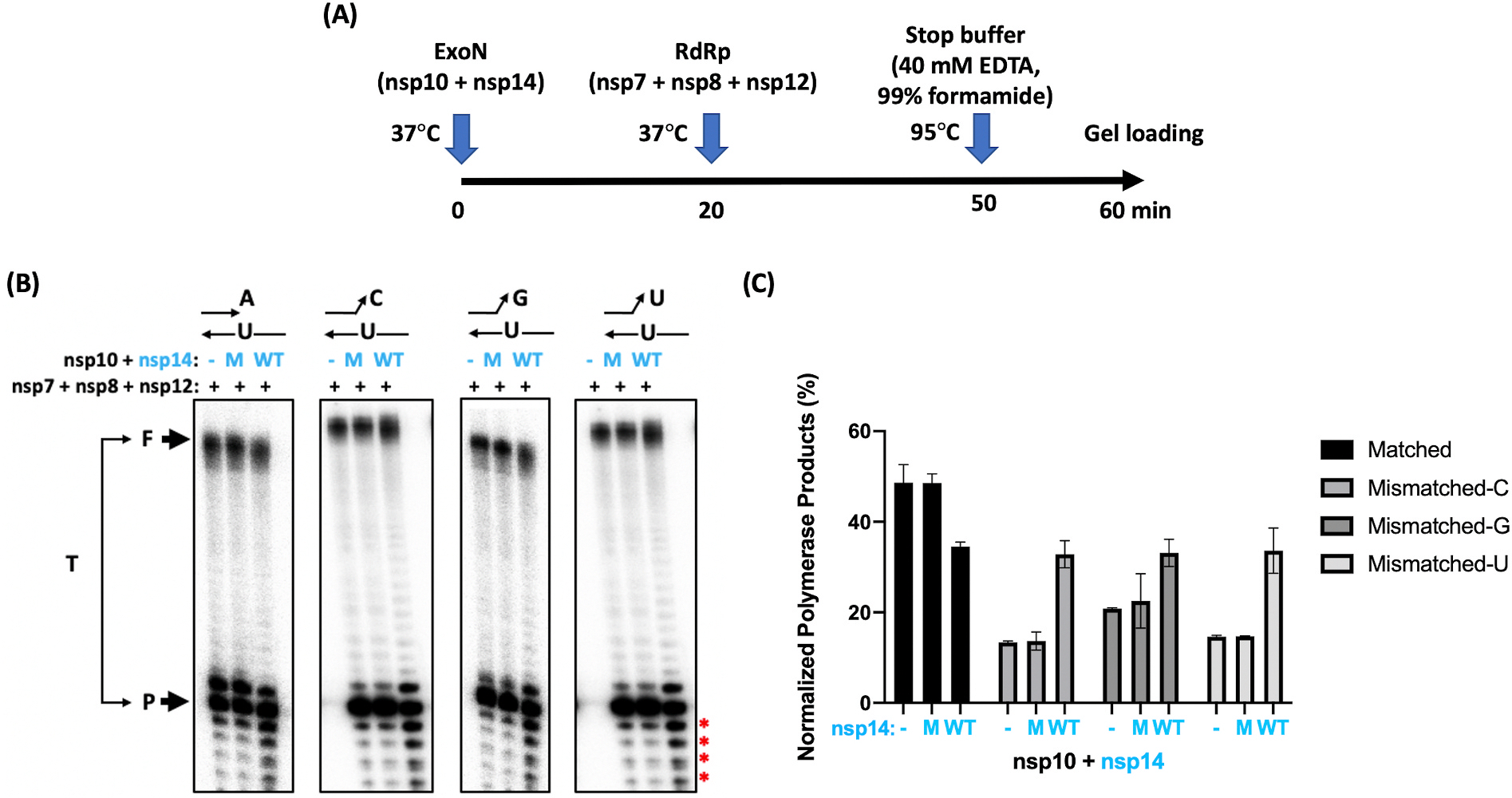
SARS-CoV-2 ExoN repairs mismatched 3′ ends of primers and rescues RdRp efficiency. (A) Experimental timeline whereby RNA primer-template sets with matched or mismatched 3′ ends on the primers were subjected to SARS-CoV-2 ExoN treatment prior to polymerization using the RdRp complex. (B) ExoN complex used to initiate RNA degradation includes nsp10 in combination with either nsp14 WT or E191A/D273A double mutant *“M”*. The amount of full-length RNA products *“F”* extended from the primers *“P”* for each reaction was normalized to the total radiation signal *“T”* quantified for respective lanes. ExoN-degraded RNA fragments were marked in red asterisks. (C) Graphical representation of normalized polymerase products extended from each ExoN-treated primer-template sets as illustrated in (B).

As observed in the mismatch primer extension assay (Fig. 3), SARS-CoV-2 RdRp exhibits reduced RNA syntheses when there is a mismatch at the 3′ end of the primer. Hence, we next tested whether the reduced RNA synthesis from the mismatched primers would be rescued when the mismatch ends were repaired by the nsp10-nsp14 complex ExoN activity. For this test, as summarized in Fig. 5A, we first pre-incubated the match or mismatched primer:templates with wild type or mutant nsp10-nsp14 complex for the 3′ end primer processing (20 min), before SARS-CoV-2 RdRp and rNTP substrates were added to synthesize the full-length RNA products (30 min), which were then subsequently analyzed. In this assay (Fig. 5B and C), there was a slight decrease in RdRp product levels when the matched primer-template (A: U) was extended following pre-exposure to WT nsp10-nsp14 complex, and possibly this decrease may result from the degradation of the total primers annealed to the template that can be extended by the RdRp. However, even with this potential loss of some primers annealed to the template by the ExoN activity, the mismatch primer extension efficiencies were noticeably rescued in the presence of ExoN (Fig. 5B and C). Possibly, following cleavage by WT nsp10-nsp14 complex, shorter but matched 3’ end RNA fragments were generated (marked as red “*“), and some of these shorter but matched primers could be extended by the RdRp, which generated more full-length products under this reaction condition. Overall, the biochemical simulation of the ExoN and RdRP reactions in Fig. 5 indicates that the nsp10-nsp14 complex can rescue the reduced RNA synthesis induced by the mismatches that are generated during the mutation synthesis process of SARS-CoV-2 RdRp.

### Mismatches generated during nucleotide misincorporation by SARS-CoV-2 RdRp are removed by ExoN cleavage:

**Fig. 6.**
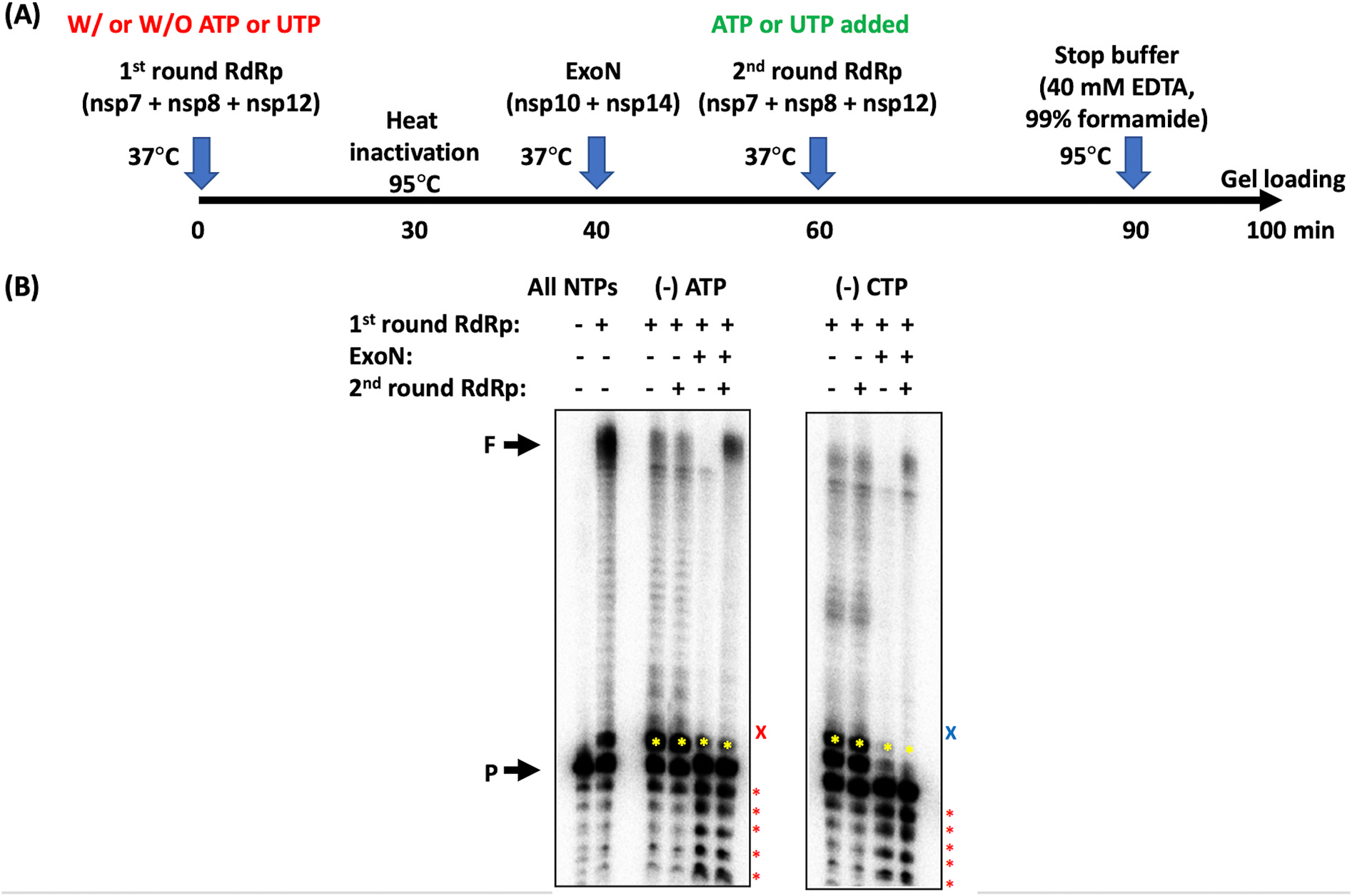
Mismatch-generating nucleotides misincorporated into RNA strands are removed by ExoN cleavage. (A) Experimental timeline whereby nucleotides misincorporation in the absence of ATP or UTP, were first induced during SARS-CoV-2 RdRp reaction, followed by ExoN cleavage of the mismatch nucleotides. The ExoN-repaired RNAs were then subjected to another round of RdRp reaction in the presence of the initially absent NTP. During the second round of RdRp reaction, fresh SARS-CoV-2 RdRp complex, MnCl_2_ and the full set of NTPs were added in similar amounts as the first RdRp reaction. (B) Gel illustration of full-length RNA products *“F”* extended from the primers *“P”* for each specific set of reactions as described. Pause sites were marked as “*” in yellow, whereas the site where the missing NTP was supposed to be incorporated was denoted as “X” (ATP in red; UTP in blue). ExoN-degraded RNA fragments were marked in red asterisks.

Finally, we tested whether the nsp10-nsp14 complex can remove mismatches generated during nucleotide misincorporation by SARS-CoV-2 RdRp. To test this, as summarized in Fig. 6A, we constructed a simulation of three sequential steps, 1) mismatch generation by SARS-CoV-2 RdRp (misincorporation reaction with biased rNTP pools, -ATP or -CTP), 2) incubation with nsp10-nsp14 complex (mismatch repair reaction), and 3) RNA synthesis by the RdRp (RNA polymerization reaction with all four rNTPs). First, the misincorporation assays (-ATP or -CTP, 1st round RdRp) gave reduced full-length product, compared with the all four NTP reaction (“F”), generating the pause sites (yellow “*“). When these misincorporation assay products were incubated further with fresh RdRp and all four NTPs (2nd round RdRp), the total product was not significantly increased, indicating the kinetic block by the initial misincorporation. When the initial misincorporation products were incubated with nsp10-nsp14 complex, all extended products were degraded. However, when these degraded products were incubated with SARS-CoV-2 RdRp and all four NTPs (2nd round RdRp), higher levels of the fully extended products were observed, compared to the products generated without the incubation with nsp10-nsp14 complex. Overall, this indicates that the nsp10-nsp14 complex successfully removed mismatches generated by nucleotide misincorporation by SARS-CoV-2 RdRp (1st round RdRp), hence generating more fully extended RNA products during the 2nd round RdRp reaction.

## 4. Discussion

During rapid RNA replication process of RNA viruses, incorrect rNTPs are often misincorporated into viral RNA genomes by viral RNA polymerases, which contributes to viral genomic mutagenesis, and if the misincorporation events frequently occur, this can contribute to their highly mutagenic nature and efficient evolution capability (Dangerfield et al., 2022; Gong, 2022). Conditions such as nucleotide pool imbalances have been known to generate DNA or RNA polymerases-induced mutations in the genomes of different viruses (Oo et al., 2020; Sabariegos et al., 2022; Shepard et al., 2019; Stapleford et al., 2015). Nucleotide misincorporation during viral DNA or RNA syntheses are among the most common outcomes of varying nucleotide availability. Frameshifts involving single-base substitution or deletion were observed when HIV-1 reverse transcription was initiated within non-equimolar dNTP concentrations (Bebenek et al., 1992). In fact, inhibition of *de novo* nucleotide biosynthesis was reported to suppress viral RNA replication, whereby viruses with low RdRp fidelity were more sensitive to changes in nucleotide pools (Chen et al., 2019; Ferron et al., 2018; Wang et al., 2011, 2016). However, there is a trade-off between replication and mutagenesis because mutation synthesis during RNA polymerization can generate kinetic delay of the RNA synthesis especially when mutation synthesis occurs at high levels. Hitherto, Nidoviruses and Arenaviruses are the only known RNA viruses which encode for ExoN that provide proofreading properties to ensure minimal genomic heterogeneity among newly synthesized RNA genomes in the viral progenies (Cruz-González et al., 2021; Hernández et al., 2022). SARS-CoV-2 which belongs to the *Nidovirale* order, possesses ExoN domain in its nsp14 (Liu et al., 2021). Working in tandem as a complex with its nsp10 cofactor, it was proposed that the nsp14 ExoN is crucial in safeguarding the fidelity of SARS-CoV-2 RdRp complex during viral replication. However, the ExoN can also play an additional role in overall viral replication kinetics by removing kinetic blocks generated by mutation synthesis caused by the error-prone RNA polymerases.

Our biochemical assays in this study demonstrated that SARS-CoV-2 RdRp complex can misincorporate and extend mismatch primers generated by the misinsertions (Figs. 1 and 3). Importantly, SARS-CoV-2 RdRp appears to efficiently extend mismatch primers, which was observed in our previous study on HIV-1 RT (Skasko et al., 2005), giving a mechanistic hint for the possibility of the error-prone nature of SARS-CoV-2 RdRp. However, the nsp10-nsp14 complex ExoN cleavage activity was shown to be able to remove mismatches (Figs. 4 and 5). Obviously, this mismatch removal activity of nsp10-nsp14 complex can provide a key proofreading mechanism in ensuring faithful replication of the viral RNA genome by counteracting the potentially error-prone nature of SARS-CoV-2 RdRp. Furthermore, our simulation shown in Fig. 6 demonstrated that the nsp10-nsp14 complex ExoN activity can elevate the levels of the full-length RNA products by clearing the kinetic blocks generated by the mutation synthesis during RNA production. This supports the idea that viral ExoN activity may also contribute to the completion of the viral replication process, especially for viruses with large viral RNA genome sizes that cannot afford kinetic blocks during viral RNA synthesis.

Apart from preserving the fidelity during viral genomic replication, the SARS-CoV-2 ExoN activity is an equally important proviral defense mechanism against antiviral agents such as remdesivir and sofosbuvir. Remdesivir is a direct acting adenosine analog that results in delayed chain termination of RNA syntheses upon incorporation by SARS-CoV-2 RdRp (Bravo et al., 2021). It exhibits potent nanomolar inhibition against SARS-CoV-2 in primary human airway epithelial cells (Pruijssers et al., 2020). Clinical trials have shown that remdesivir treatments effectively shortened the recovery time of hospitalized COVID-19 patients, whereas early intervention with this drug resulted in decreased risk of death or hospitalization among non-hospitalized individuals (Garibaldi et al., 2021; Gottlieb et al., 2022). On the other hand, sofosbuvir which is commonly used to treat hepatitis C, is a uridine analog that terminates viral RNA transcription when being incorporated into the growing RNA strand (Xu et al., 2017). A recent clinical trial report showed that treatment with sofosbuvir in combination with velpatasvir resulted in more rapid SARS-CoV-2 viral clearance and limited disease progression among COVID-19 patients (Messina et al., 2022).

Active triphosphate forms of remdesivir and molnupiravir which were incorporated by SARS-CoV-2 RdRp into the viral RNA, were shown to be effectively excised by the viral ExoN complex (Wang et al., 2022b). Viral RNA containing sofosbuvir triphosphate was in fact found to be more resistant against ExoN cleavage in comparison with that of remdesivir triphosphate (Jockusch et al., 2020). While this would impair the antiviral potency of either drug, on a broader perspective, this also contributes to the development of viral resistance towards therapeutic regiments involving those inhibitors (Wang et al., 2022c). Hence, this has led to new antiviral strategies and drug combinations constantly being developed and tested to combat this disease. *In vitro* biochemical and biological assays have demonstrated that combinations of RdRp and ExoN inhibitors resulted in enhanced inhibitory potency against SARS-CoV-2 (Wang et al., 2022b). The underlying feature of this drug combination therapy is to conserve the RdRp-inhibiting effects of molecules such as nucleoside analogs via the suppression of ExoN cleavage activity.

In conclusion, we have utilized biochemical approaches to simulate the enzymatic processes that occur during mutation syntheses by SARS-CoV-2 RdRp and its repair by the ExoN. However, it is important to note that like most reported biochemical assays, our simulations presented in this study may not represent the actual physiological conditions in which infectious SARS-CoV-2 replicates during an infection. For instance, concentrations of the divalent cofactors used in this study namely Mg^2+^ and Mn^2+^, may not be as abundantly available within the human system. Nonetheless, the amounts used were necessary to ensure the efficient functionality of respective viral enzymes in our *in vitro* cell-free simulation. While in general, plenty of emphases have been placed on identifying and deciphering antiviral drugs and prophylactic vaccines for SARS-CoV-2, it is also important to have more in-depth knowledge on the molecular and mechanistic levels of the virus replication. The mechanistic analyses of both crucial viral enzymatic properties in this study further enhanced the understanding on SARS-CoV-2 mutagenic nature during viral replication and the role of its proofreading mechanism in ensuring genomic replication fidelity.

## Supplementary Material

Supplementary data

## Data Availability

All data are contained within the manuscript.

## Appendix A. Supplementary data

Supplementary data to this article can be found online at https://doi.org/10.1016/j.virol.2024.110255.
