# Supplementary figures and images for: Biochemical simulation of mutation synthesis and repair during SARS-CoV-2 RNA polymerization
Source: Virology. Author manuscript; Available in PMC 2026 Jul 18. (PMC13380264; doi:10.1016/j.virol.2024.110255)

Supplement Figures

Figure S1


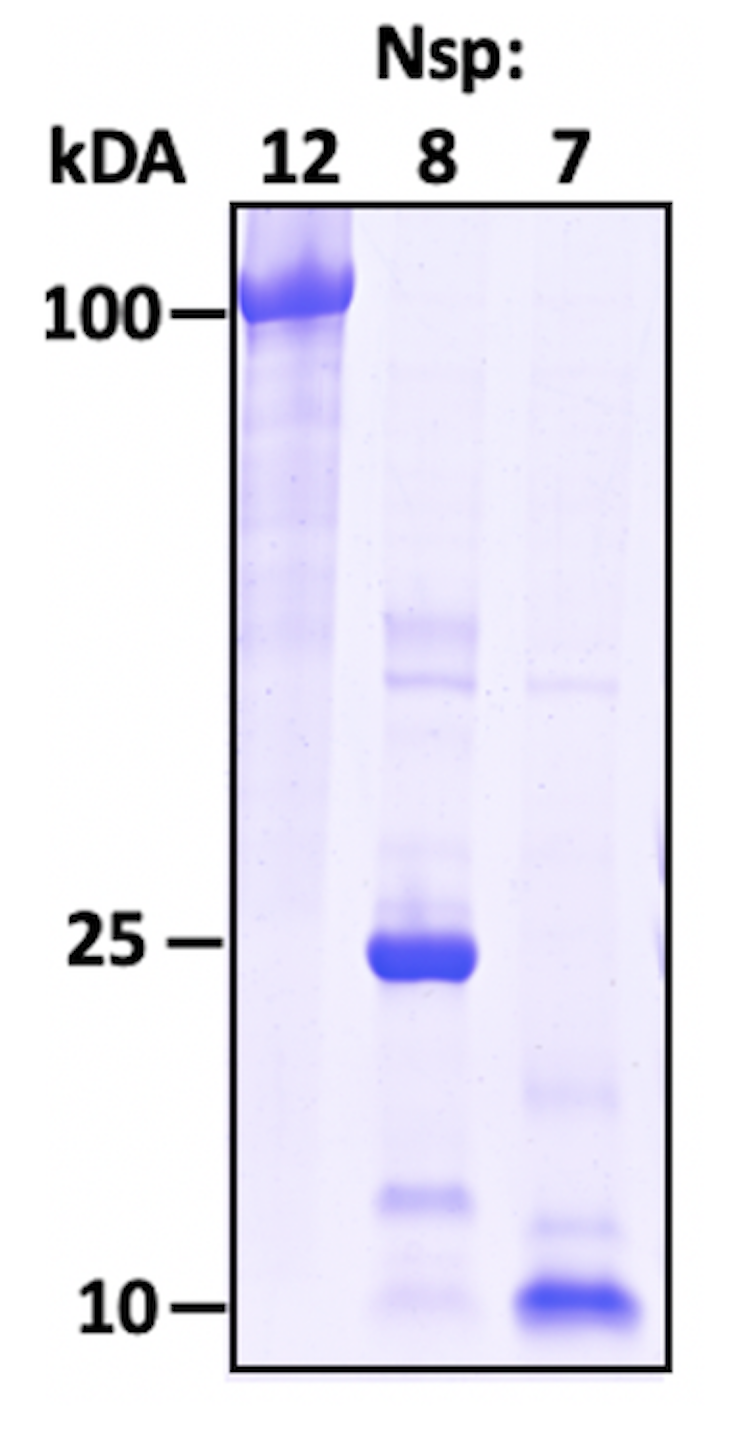


Figure S2


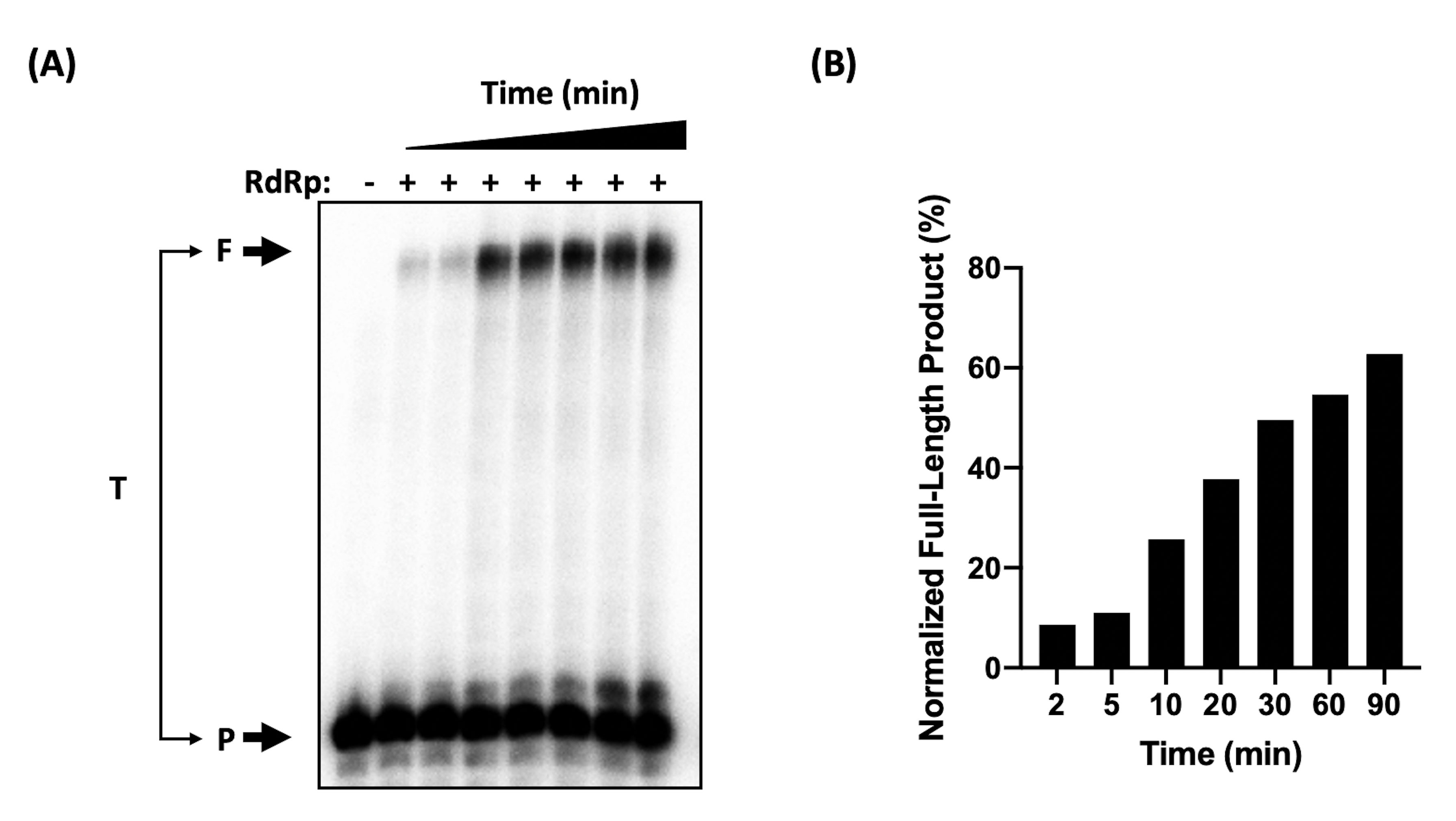


Figure S3


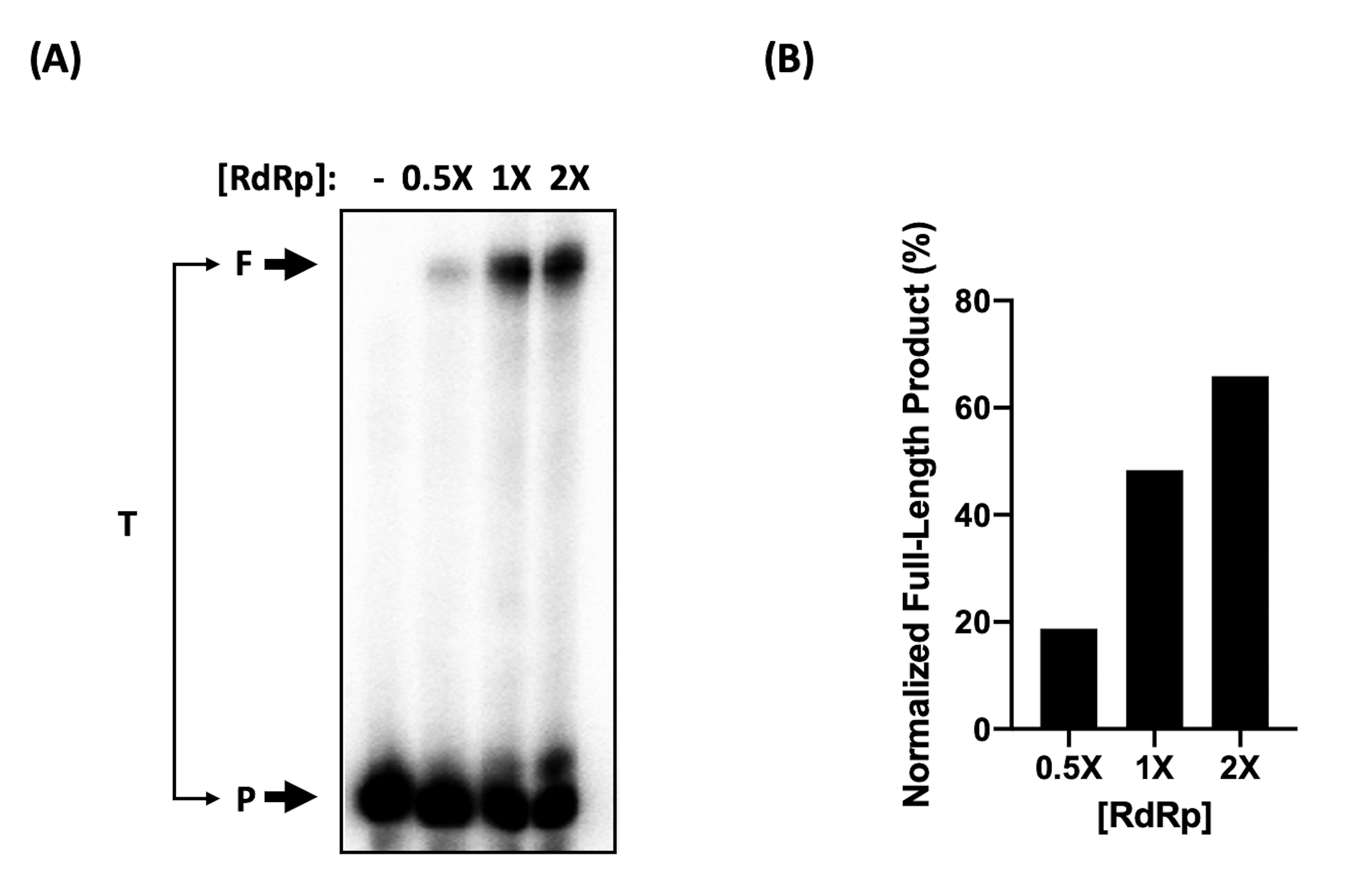

Supplement: Supplementary data [file NIHMS2187271-supplement-Supplementary_data.docx]
